# Therapeutic advancement of chronic lymphocytic leukemia

**DOI:** 10.1186/1756-8722-5-55

**Published:** 2012-09-16

**Authors:** Kang Lu, Xin Wang

**Affiliations:** 1 Department of Hematology, Provincial Hospital Affiliated to Shandong University, No. 324 Jingwu Road, Jinan, Shandong, 250021, P R China; 2 Department of Diagnostics, Shandong University School of Medicine, Jinan, Shandong, 250012, P. R. China

**Keywords:** Chronic lymphocytic leukemia, Treatment, Monoclonal antibodies

## Abstract

Despite the combinations of chemotherapy with monoclonal antibodies have further improved response rates, chronic lymphocytic leukemia (CLL) remains an incurable disease with an extremely variable course. This article reviews the ongoing clinical advances in the treatment of CLL in both previously untreated and relapsed disease and focuses on the benefit of different therapeutic strategies, the most effective therapy combinations and the potential activity of novel agents. Novel agents and combination therapies have been investigated by several studies in both the upfront and relapsed setting, particularly for patients with 17p deletion, TP53 mutation and fludarabine-refractory CLL. While these agents and combination therapies have improved initial response rates, ongoing studies are continued to determine and improve the efficacy and safety. Despite advancements in the treatment of CLL have led to high response rates, allogeneic hematopoietic stem cell transplantation (allo-HSCT) remains the only curative option and reduced-intensity conditioning (RIC) allo-HSCT must be strongly considered whenever feasible. As such, ongoing studies of these agents and other novel approaches in clinical development are needed to expand and improve treatment options for CLL patients.

## Introduction

Chronic lymphocytic leukemia (CLL) remains an incurable disease with an extremely variable course; survival after diagnosis can range from months to decades [1]. Genomic features such as mutational status of immunoglobulin heavy chain variable region genes (IGHV), ataxia telangiectasia mutated (ATM) and TP53 tumor suppressor genes, β2-microglobulin, zeta-chain-associated protein kinase 70 (ZAP70) expression, interphase cytogenetics, and complex karyotype on metaphase cytogenetics, provide further differentiation of disease prognosis [2]. As a result, therapy must be flexible and tailored for different patient groups [3]. With the improvement of its associated prognostic and therapy response factors, the treatment of CLL has dramatically changed. Focusing on the benefit of different therapeutic strategies, this article reviews recent advancements, the most effective therapy combinations and potential activity of novel agent in the treatment of CLL.

### Chemotherapy

Although chlorambucil gave lower overall response rates (ORRs) and a shorter progression-free survival (PFS) compared with the purine analogue fludarabine [4], chlorambucil administered as front-line therapy to elderly CLL patients ≥65 years proved as effective in sustaining remissions as fludarabine (Table 1) [5]. Bendamustine, a cytotoxic hybrid of an alkylating agent and a purine analog, was introduced for the treatment of CLL in 2008 [6]. Based on the results of an open-label phase 3 trial in 319 previously untreated CLL patients, single-agent bendamustine has shown improved ORR and complete response (CR) rate (68% and 31%) when compared with chlorambucil (31% and 2%) [7]. The median PFS was 21.6 months with bendamustine and 8.3 months with chlorambucil (P < 0.0001) (Table 1). A retrospective chart review showed that bendamustine, either alone or with rituximab, provided meaningful response rates and was generally well tolerated in patients ≥70 years old with CLL [8].

**Table 1:** Phase 3 trials of chemotherapy in previously untreated CLL patients

**Reference (year)**	**Regimen**	**Number of patients**	**Median age (years)**	**CR,%**	**ORR,%**	**Median PFS (months)**	**OS**
Rai, 2000 [4]	Chlorambucil	181	62	4	37	14	Median 56 m
	Fludarabine	170	64	20	63	20	Median 66 m
Eichhorst, 2009 [5]	Chlorambucil	100	70	0	51	18	Median 64 m
	Fludarabine	93	71	7	72	19	Median 46 m
Knauf, 2009 [7]	Chlorambucil	157	66	2	31	8.3	NR
	Bendamustine	162	63	31	68	21.6	NR
Eichhorst, 2006 [9]	Fludarabine	164	59	7	83	20	81% at 3 y
	FC	164	58	24	94	48	80% at 3 y
Catovsky, 2007 [10]	Fludarabine	194	64	15	80	23	52% at 5 y
	FC	196	65	38	94	43	54% at 5 y
	Chlorambucil	387	65	7	72	20	59% at 5 y
Flinn, 2007 [11]	Fludarabine	137	61	5	60	19	80% at 2 y
	FC	141	61	23	74	32	79% at 2 y
Robak, 2010 [13]	CC	192	58	47	88	28	62.4% at 4 y
	FC	203	59	46	82	27	60.6% at 4 y
Robak, 2011 [15]	Cladribine	174	61	21	78	27.2	Median 45 m
	CC	171	62	29	83	22.4	Median 48 m
	CMC	163	59	36	80	25.6	Median 46 m

Abbreviations: CC, cladribine, cyclophosphamide; CMC, cladribine, cyclophosphamide, mitoxantrone; CR, complete response; FC, fludarabine, cyclophosphamide; NR, not researched; ORR, overall response rate; OS, overall survival; PFS, progression-free survival.

Three randomized trials in untreated CLL patients comparing fludarabine and cyclophosphamide (FC) with fludarabine monotherapy showed superior CRs, ORRs and PFS for the FC arm [3,9-11], while a analysis of the subgroup of patients without high-risk genetic deletions showed superior overall survival (OS), in these patients comparing FC with fludarabine monotherapy [12]. A randomized phase 3 trial showed that cladribine and fludarabine in combination with cyclophosphamide are equally effective and safe first-line regimens for progressive CLL. However, both combinations have unsatisfactory activity in patients with 17p13 (TP53 gene) deletion (Table 1) [13]. One trial of cladribine (C) versus cladribine and cyclophosphamide (CC) versus cladribine, cyclphosphamide plus mitoxantrone (CMC) showed superior CRs for the CMC arm over C but not the CC arm [14]. However, long term results for these patients confirmed that cladribine alone, CC and CMC regimens produced comparable PFS and OS in previously untreated progressive CLL (Table 1) [15].

### Monoclonal antibodies

#### Rituximab

As the first approved therapeutic antibody for the treatment of cancer, rituximab used in CLL has been extensively explored. Numerous studies combining rituximab with other therapies have been pursued, as summarized in Table 2. In a randomized phase 2 study, a total of 104 patients were enrolled. Fludarabine with concurrent rituximab (n = 51) was compared to fludarabine with sequential rituximab (n = 53) in untreated patients with CLL [16]. After a median follow-up of 117 months (range, 66 to 131 months), the median PFS and OS times for the treatment groups were similar, with an overall estimated median PFS of 42 months (95% CI, 31 to 46 months) and median OS of 85 months (95% CI, 71 to 95 months) [17]. These long-term data support fludarabine plus rituximab as one acceptable first-line treatment for symptomatic patients with CLL.

**Table 2:** Selected trials of chemoimmunotherapy with rituximab in CLL patients

**Reference (year)**	**Regimen**	**Phase**	**Number of patients**	**Treatment status**	**CR,%**	**ORR,%**	**Median PFS (months)**	**Median OS (months)**
Woyach, 2011 [17]	FR (C)	2	51	Untreated	47	90	42	85
	FR (S)		53	Untreated	28	77		
Kay, 2007 [18]	PCR	2	64	Untreated	41	91	32.6	Not Rep
Kay, 2010 [19]	PR	2	33	Untreated	27	76	12	Not Rep
Reynolds, 2011 [20]	PCR	3	92	20% Pretreated	7	49	NR	NR
	FCR		92	20% Pretreated	14	59	NR	NR
Wierda, 2011 [21]	FBR	1/2	14	Untreated	36	93	Not Rep	Not Rep
Bosch, 2011 [23]	R-FCM	2	72	Untreated	82	93	Not Rep	Not Rep
Jenke, 2011 [24]	CHOP-R	2	26	Pretreated, F-ref	0	54	11	27
			15	Pretreated, RT	7	67	15	27
			15	Pretreated, AIC	0	82	14	50

Abbreviations: AIC, autoimmune cytopenia; CHOP-R, cyclophosphamide, adriamycin, vincristine and prednisone plus rituximab; CR, complete response; FBR, fludarabine, bendamustine, and rituximab; FR (C), fludarabine, rituximab, concurrent; FR (S), fludarabine, rituximab, sequential; FCR, fludarabine, cyclophosphamide, rituximab; F-ref, fludarabine-refractory; NR, not researched; Not Rep, not reported; ORR, overall response rate; OS, overall survival; PCR, penostatin cyclophosphamide, rituximab; PFS, progression-free survival; R-FCM, rituximab, fludarabine, cyclophosphamide, mitoxantrone; RT: Richter’s transformation.

The combination of pentostatin, cyclophosphamide, and rituximab (PCR) achieved an ORR >90%, with >40% CR in patients with untreated CLL [18]. Moreover, the findings of a phase 2 trial suggested that increasing the dose of the purine nucleoside analogue did not eliminate the need for cyclophosphamide in chemoimmunotherapy for the treatment of CLL [19]. A phase 3 trial compared the combination of fludarabine, cyclophosphamide, and rituximab (FCR) and PCR in previously untreated or minimally treated B-cell CLL [20]. The infection rate (FCR/PCR) was 31%/36%, 12 (14%)/6 (7%) patients achieved CR; the ORR including CR, partial response (PR), and nodular PR (nPR) was 59%/49%.

A phase 1/2 trial of fludarabine, bendamustine, and rituximab (FBR) chemoimmunotherapy for previously treated patients with CLL indicated that the FBR regimen was tolerated up to the highest bendamustine dose evaluated with significant efficacy [21]. In another study, retherapy with bendamustine, mitoxantrone and rituximab in patients with relapsed/refractory CLL and indolent lymphomas also achieved high response rates [22]. Another chemoimmunotherapies with rituximab in CLL patients were also studied in recent trials [23,24].

#### Alemtuzumab

As a recombinant monoclonal antibody that targets the CD52 cell-surface antigen, alemtuzumab significantly improved PFS, time to alternative treatment, ORR and CR, and minimal residual disease-negative remissions in previously untreated CLL patients, compared with chlorambucil (Table 3) [25]. Furthermore, alemtuzumab demonstrated significant activity in patients with bulky nodes, as evidenced by an ORR in patients with lymph nodes ≥5 cm of 76% in alemtuzumab-treated patients versus 44% in chlorambucil-treated patients (P = 0.0125). Alemtuzumab also showed a promising safety profile coupled with satisfactory effectiveness in poor prognosis CLL [26,27]. The combination of alemtuzumab and oral dexamethasone showed high response rates in elder patients with ultra high-risk CLL, with promising preliminary findings for PFS and OS. However, the improved initial response by adding dexamethasone did not seem to translate into improved long-term results, when compared to the preceding CLL2H study with single agent alemtuzumab (Table 3) [28].

**Table 3:** Selected trials of alemtuzumab monotherapy and chemoimmunotherapy in CLL patients

**Reference (year)**	**Regimen**	**Phase**	**Number of patients**	**Treatment status**	**CR,%**	**ORR,%**	**Median PFS (months)**	**Median OS (months)**
Hillmen, 2007 [25]	Alemtuzumab	3	149	Untreated	24	83	14.6	NR
	Chlorambucil		148	Untreated	2	55	11.7	NR
Gritti, 2012 [27]	Alemtuzumab	2	18	F-ref	8	44	10.3	29.1
Stilgenbauer, 2011 [28]	AD	2	30	Untreated, del(17p)	20	97	16.9	>24 , NR
			17	Relapse, del(17p)	0	76	10.4	15
			40	F-ref	5	70	8.4	12
Geisler, 2011 [29]	AFC	3	129	Untreated, high-risk	57	88	37	NR
	FC		133	Untreated, high-risk	45	80	31	NR
Badoux, 2011 [30]	CFAR	2	80	Refractory /relapse	29	65	10.6	N/A
Zent, 2011 [31]	PAR	2	19	Refractory /relapse	32	74	7	23

Abbreviations: A, alemtuzumab; AD, alemtuzumab and dexamethasone; AFC, alemtuzumab plus fludarabine and cyclophosphamide; CFAR, fludarabine, cyclophosphamide, alemtuzumab and rituximab; CR, complete response; FC, fludarabine and cyclophosphamide; F-ref, fludarabine-refractory; N/A, not applicable; NR, not researched; Not Rep, not reported; ORR, overall response rate; OS, overall survival; PAR, pentostatin, alemtuzumab, and rituximab.

Alemtuzumab based chemoimmunotherapy has also shown good responses in relapsed/refractory disease (Table 3). An early analysis of the randomized phase 3 CLL trial recently indicated that chemoimmunotherapy with low-dose subcutaneous alemtuzumab plus oral fludarabine and cyclophosphamide was safe and induced more and deeper CRs in untreated patients with high-risk CLL than chemotherapy with FC alone [29]. Good response rates in highly pretreated high-risk group of patients were indicated in a phase 2 study of alemtuzumab plus FCR (CFAR), although there was no benefit in survival outcomes [30]. Another study also indicated that pentostatin, alemtuzumab, and low dose rituximab was effective therapy for relapsed/refractory CLL/small lymphocytic lymphoma (SLL) [31].

Final results of a phase 2 study in previously untreated elderly CLL patients indicated that three courses of FC only yielded a rather high response rate and a short (8 weeks) alemtuzumab consolidation course could thereafter be administered safely, leading to a 52% rate of PR to CR switches, a high proportion of patients with undetectable blood MRD after the end of treatment and durable responses. Overall, 26 patients were in CR after the whole treatment strategy accounting for 51% of the entire cohort [32]. In another study, Alemtuzumab consolidation for residual disease after treatment with high-dose methylprednisolone plus rituximab (HDMP-R) was well tolerated and effective in patients with CLL [33]. However, alemtuzumab consolidation did not improve outcome for CLL patients with high risk genomic features on successive Cancer and Leukemia Group B (CALGB) trials [34].

#### Ofatumumab

Ofatumumab is a fully humanized CD20 monoclonal antibody that targets an epitope different from the epitope targeted by rituximab. Based on the interim analysis of the pivotal international clinical trial, which included data from 138 CLL patients refractory to fludarabine and alemtuzumab (FA-ref) and refractory to fludarabine but did not receive treatment with alemtuzumab due to bulky disease (BF-ref), ofatumumab had been approved by the FDA for patients with CLL refractory to fludarabine and alemtuzumab in October 2009 [35]. At this interim analysis, the ORR (primary endpoint) with single-agent ofatumumab was 58% (99% CI: 40, 74) in the FA-ref group and 47% (99% CI: 32, 62) in the BF-ref group. The final results for the primary endpoint of this study in 206 enrolled patients indicated that the ORR was 51% for the FA-ref group and 44% for the BF-ref group (Table 4) [36]. In another phase 2 trial of ofatumumab for older patients and patients who refused fludarabine-based regimens with previously untreated CLL or SLL, 13 patients (44%) achieved an objective response (CR, 0; PR, 13); 16 (53%) patients had stable disease (SD); 1 patient (3%) had progressive disease (PD) (Table 4) [37].

**Table 4:** Phase 2 trials of ofatumumab monotherapy and chemoimmunotherapy in CLL patients

**Reference (year)**	**Regimen**	**Number of patients**	**Treatment status**	**CR,%**	**ORR,%**	**Median PFS (months)**	**Median OS (months)**
Wierda, 2010 [36]	Ofatumumab	95	FA-ref	0	51	5.5	14.2
		111	BF-ref	2	44	5.5	17.4
Flinn, 2011 [37]	Ofatumumab	42	Untreated	0	44	NR	NR
Wierda, 2011 [38]	O-FC (500 mg)	31	Untreated	32	77	NR	NR
	O-FC (1000 mg)	30	Untreated	50	73	NR	NR
Shanafelt, 2011 [39]	PCO	33	Untreated	45	94	NR	NR

Abbreviations: BF-ref, fludarabine-refractory CLL with bulky (>5 cm) lymphadenopathy; CR, complete response; FA-ref, fludarabine- and alemtuzumab-refractory; NR, not researched; O-FC (500 mg), 500 mg ofatumumab combined with fludarabine and cyclophosphamide; O-FC (1000 mg), 1000 mg ofatumumab combined with fludarabine and cyclophosphamide; ORR, overall response rate; OS, overall survival; PCO, pentostatin, cyclophosphamide and ofatumumab.

Front-line ofatumumab-based chemoimmunotherapy appeared to be well-tolerated in patients with CLL. An international phase 2 trial investigated the efficacy and safety of 2 dose levels of ofatumumab combined with fludarabine and cyclophosphamide (O-FC) in previously untreated patients with CLL. In this trial, 61 patients were randomized to the ofatumumab 500 mg (n = 31) or 1000 mg (n = 30) dose cohorts. The CR rate was 32% for the 500 mg and 50% for the 1000 mg cohort, and the ORR was 77% and 73%, respectively. The most frequent Common Terminology Criteria grade 3–4 investigator-reported adverse events were neutropenia (48%), thrombocytopenia (15%), anemia (13%), and infection (8%) (Table 4) [38]. Another clinical trial has been initiated to study the effect of ofatumumab in combination with pentostatin and cyclophosphamide (PCO) for patients with previously untreated CLL (Table 4) [39]. Compared to the historic experience with rituximab-based chemoimmunotherapy, ofatumumab-based chemoimmunotherapy appeared to have less hematologic toxicity and improved efficacy.

#### Lumiliximab

Lumiliximab is a macaque-human primatized monoclonal antibody that targets the CD23 antigen. In a phase 1 trial, investigators found that lumiliximab was well tolerated but showed minimal activity [40]. In a phase 1/2 trial, FCR plus lumiliximab resulted in an OR rate of 65% and a CR rate of 52%, and the toxicity of the combination appeared no different from that which was previously reported with FCR in treatment of relapsed CLL [41]. However, a phase 3 study comparing FCR to FCR plus lumiliximab in relapsed CLL showed no benefit in terms of improved response rate of PFS with the addition of lumiliximab to FCR [42].

#### Obinutuzumab

Obinutuzumab (GA101) is a humanized, third generation, type II CD20 IgG1 antibody with a glycoengineered Fc region [43]. It exhibits enhanced antibody-dependent cellular cytotoxicity and superior caspase-independent apotosis induction in comparison with classic type I CD20 antibodies, such as rituximab. Modification of elbow hinge sequences within the antibody variable framework regions resulted in a strong apoptosis-inducing activity of obinutuzumab. Complement and antibody-dependent cellular cytotoxicity are believed to be the major effector mechanisms of obinutuzumab in whole blood assays [44]. 13 patients with relapsed or refractory disease were enrolled in a phase 1 study of obinutuzumab. The results showed that the ORR was 62% (1 CRi, 7 PRs, and 5 SDs) with no clear dose relationship established [45].

### Immunomodulatory drugs

The encouraging antitumour activity of thalidomide in various malignant disorders led to development of subsequent analogues. The immunomodulatory agents lenalidomide and pomalidomide have shown promising antineoplastic activity in various tumor types [46,47]. Lenalidomide has demonstrated clinical efficacy in CLL through various mechanisms [48]. The results of a recent study showed that lenalidomide therapy was well tolerated and induced durable remissions in the population of elderly, symptomatic patients with CLL [49]. In this study, sixty patients with CLL, which were 65 years of age and older, received treatment with lenalidomide. At a median follow-up of 29 months, 53 patients (88%) are alive and 32 patients (53%) remain on therapy. Estimated 2-year PFS was 60%. The ORR to lenalidomide therapy was 65%, including 10% CR, 5% CR with residual cytopenia, 7% nPR, and 43% PR. Neutropenia was the most common grade 3 or 4 treatment-related toxicity observed in 34% of treatment cycles. Major infections or neutropenic fever occurred in 13% of patients.

A phase 2, 2-stage study was designed to evaluate the combination of lenalidomide and rituximab for the initial treatment of patients with CLL [50]. 40 patients enrolled into arm A (age under 65), and 29 into arm B (age 65 or older). The median age on arm A was 57 years (range 45–64) and arm B 70 years (range 65–80). The ORR to therapy for arm A was 94%, with 20% achieving a CR and 17% a nPR. The ORR for arm B was 77% with 9% achieving a CR. Arm A patients had median follow-up of 17 months with an estimated median PFS of 19 months. Arm B had a median follow-up of 7 months, with an estimated 85% remaining progression free at 7 months. Another phase 2 study of lenalidomide and rituximab in patients with relapsed or refractory CLL indicated that the combination of lenalidomide and rituximab led to durable responses in patients with relapsed and refractory CLL and was active also in patients with 17p deletion [51]. The combination therapy with ofatumumab and lenalidomide in patients with relapsed CLL was also conducted in a phase 2 trial. 5 patients (15%) achieved CR (including 1 CRi) and 17 patients (50%) PR, for an ORR of 65% [52].

Lenalidomide-based consolidation for CLL patients receiving first-line chemoimmunotherapy induction appeared to improve the quality of response and prolonged time to retreatment [53]. Based on early experience, lenalidomide consolidation after chemotherapy could further improve responses in 27% of patients with CLL. Elimination of MRD was seen in 12% of patients treated [54].

### High-Dose Methylprednisolone

High-dose methylprednisolone (HDMP) has proved to be an active treatment in patients with relapsed/refractory CLL, including those with unfavorable cytogenetic features by numerous studies [55-57]. A phase 2 study combining rituximab with HDMP as a salvage regimen for the treatment of patients with fludarabine-refractory CLL showed an ORR of 93%, with a CR rate of 36% [58]. Another report from the same group showed a similar efficacy in the front-line setting: an ORR of 96%, with a CR rate of 32% using a reduced number of days of methylprednisolone [59]. Although a lower ORR of 78% (22% CR) was reported by a larger single-institution review of 37 patients who were treated with the same HDMP plus rituximab combination, a high response rate was showed for those with high-risk cytogenetic abnormalities, including ORRs of 55.6% and 66.7% for those with 17p deletion and 11q deletion, respectively [60].

Another study evaluated the efficacy and safety of dose-dense HDMP plus rituximab in patients with high-risk CLL. 29 patients with relapsed or progressive CLL with adverse cytogenetics (17p deletion, TP53 mutation, 11q deletion, and/or trisomy 12) and/or progression within 12 months of fludarabine treatment were included. The ORR was 62%, and 28% of patients had SD. In 13 patients with 17p deletion/TP53 mutation, ORR was 69%. After 22 months, the median PFS and OS were 12 and 31 months, respectively. The most frequent toxicity was hyperglycemia, and three deaths occurred in the study [61]. Combination of HDMP and ofatumumab was also demonstrated as an effective salvage treatment for heavily pretreated, unfit or refractory patients with CLL [62].

### Cyclin-dependent kinase (CDK) inhibitors

As a broad CDK inhibitor, flavopiridol (alvocidib) could induce apoptosis in human CLL cells and is independent on p53 ways. Flavopiridol has demonstrated activity in patients with relapsed CLL, including those with high-risk genomic features and bulky lymphadenopathy [63-65]. Outcomes of refractory CLL patients in two age categories (≥70, <70 years) treated with single-agent flavopiridol indicated that flavopiridol treatment of patients aged 70 or older with refractory or relapsed CLL was a feasible therapeutic approach, and may have similar efficacy relative to younger patients. No significant difference between older and younger patients was observed in response rates (43% versus 47%) or median PFS (8.7 versus 9.9 months, *P* > 0.80). Although median OS was worse in older patients (2.1 versus 2.4 years, *P =* 0.02), when adjusted for other factors, this difference was no longer significant (*P* > 0.10). With exception to infections (older = 29% versus younger = 62%), no significant association with toxicity was observed [66].

Activity of combined flavopiridol and lenalidomide in patients with cytogenetically high risk CLL was observed in a phase 1 trial. The results showed that the combination of flavopiridol and lenalidomide was well tolerated without increased risks of tumor lysis syndrome or tumor flare, with significant activity in patients with bulky, cytogenetically high-risk CLL. In 23 evaluable patients who completed 1 or more cycles of combined lenalidomide and flavopiridol, PRs were observed in 13 patients (57%). 6 patients were able to proceed to allogeneic transplant after 1–3 cycles, and 4 of these patients remain in remission. Median PFS and OS are 7 months (range 0–24 months; 95% CI 5, 11) and 23 months (range 0–27 months; 95% CI 13, 27), respectively [67].

Other related CDK inhibitors, such as dinaciclib (SCH 727965), BMS-387032 (SNS-032), sunitinib and sorafenib are being investigated in patients with relapsed or refractory CLL. In a phase 1 trial, dinaciclib appeared to have a similar response rate but less toxicity than flavopiridol in patients with relapsed or refractory CLL [68].

### Bcl-2 inhibitors

Navitoclax (ABT-263) is a small-molecule BH3 mimetic that potently inhibits BCL-2, BCL-xL, and BCL-w and is able to induce apoptosis in primary CLL cells. In a phase 1/2a trial in patients with relapsed or refractory CLL, 90% patients showed at least a 50% decrease in absolute lymphocyte count, and the ORR was 35%, all PRs. The median treatment duration was 7 months, with median PFS and time to progression of 25 months. Furthermore, the PFS was similar in fludarabine-refractory and fludarabine-sensitive patients. However, significant toxicity of thrombocytopenia may limit the use of navitoclax in heavily pretreated fludarabine-refractory CLL patients [69,70]. Combination study has been conducted to examine whether navitoclax could be used safely in combination with FCR or bendamustine plus rituximab (BR) for treatment of patients with CLL. Of the 16 patients assessed in Arm B (BR), 6 achieved CR, 7 PR, 2 SD and 1 with PD. The ORR was 81% (13/16). In this arm, 3/5 patients with 17p deletion achieved PR. Of the 4 patients assessed in Arm A (FCR), 2 achieved PR, 1 SD and 1 with PD. The combination of navitoclax with BR appeared well-tolerated and to have anti-tumor activity [71]. Other Bcl-2 inhibitors included oblimersen, gossypol (AT-101), obatoclax, SPC2996 are also in investigational phases and further studies with these agents are warranted [72-75].

### Kinase inhibitors of B-cell receptor (BCR) signaling pathways

#### Phosphatidylinositol-3-kinase (PI3K) inhibitors

In lymphocytes, the PI3K isoform p110δ (PI3Kδ) transmits signals from surface receptors, including the B-cell receptor (BCR). GS-1101 (CAL-101), an isoform-selective inhibitor of PI3Kδ that inhibits PI3K signaling, which induces apoptosis of CLL cells and reduces interactions that retain CLL cells in protective tissue microenvironments in vitro, displays clinical activity in CLL, causing rapid lymph node shrinkage and a transient lymphocytosis [76]. A phase 1 study of GS-1101 in 37 patients with relapsed or refractory CLL was reported [77]. GS-1101 reduced lymphadenopathy in all of the patients, and 91% achieved a lymph node response (≥50% reduction in target nodal lesions). The ORR was 33% (all PRs) and the median duration of response had not been reached. 75% of patients with CLL-related thrombocytopenia had either an improvement to >100,000/μL or a >50% increase from baseline. Another phase 1 trial studied GS-1101 in combination with rituximab and/or bendamustine in 27 patients with previously treated CLL [78]. The results indicated that GS-1101 offered major and rapid reductions in lymphadenopathy. Recently, the preliminary data from a phase 1 trial suggested that SAR245408, an oral pan-PI3K inhibitor, was generally well tolerated in heavily pretreated relapsed/refractory CLL [79].

#### Bruton tyrosine kinase (Btk) inhibitors

Ibrutinib (PCI-32765), a specific inhibitor of Btk, can disrupt several signaling pathways involved in tumor microenvironment interactions, induce apoptosis and inhibit cellular migration and adhesion in malignant B-cells [80]. An early analysis of the phase 1b/2 study PCYC-1102 showed ibrutinib to be highly active and tolerable in patients with CLL [81]. Nodal response was seen in 89% of patients with lymphadenopathy, with an increase in absolute lymphocyte count in 75%. At a median follow-up of 4 months, the ORR was 44% (39% PRs, 5% CRs) and 4 of 12 patients with deletion 17p had responded, suggesting activity in this subgroup. Longer-term follow-up of this multicenter phase 1b/2 trial has been recently reported [82]. Results of this analysis indicated that ibrutinib was well tolerated and was associated with high rates of 6-month PFS in relapsed or refractory CLL/ SLL. Grade 1 or 2 diarrhea, fatigue, nausea, and ecchymosis have been the most frequently reported adverse events. Serious adverse events have occurred in 38% of patients. ORR in the 420 mg cohort was 48% with 6.2 months median follow-up and 70% with 10.2 months median follow-up. ORR in the 840 mg cohort was 44% at 6.5 months median follow-up. An additional 19%, and 35% of patients in these cohorts, respectively, had a nPR with residual lymphocytosis.

#### Spleen tyrosine kinase (Syk) inhibitors

Syk is a protein tyrosine kinase that couples BCR activation with downstream signaling pathways, promoting cell activation and migration. In CLL, Syk could be activated by external signals from the tissue microenvironment. Fostamatinib disodium (R788) is the first clinically available oral Syk inhibitor. A multicenter phase 1/2 clinical trial of fostamatinib disodium in patients with recurrent B-cell NHL was reported [83]. The 11 patients with CLL/SLL enrolled in this study achieved an ORR of 55% (all PRs) and a PFS of 6.4 months. Common toxicities included diarrhea, fatigue, cytopenias, hypertension, and nausea. Preclinical studies of other Syk inhibitors such as R406 and two highly selective Syk inhibitors (PRT318 and P505-15) demonstrated responses in CLL cells supporting the development of a novel and active therapeutic approach for CLL and other selected B-cell malignancies [84,85].

#### Lyn tyrosine kinase inhibitors

Dasatinib, a tyrosine kinase inhibitor originally developed as a pan-Src kinase inhibitor, can inhibit Lyn kinase (a Src-family kinase) and lead to apoptosis of the CLL cells in vitro. A phase 2 clinical trial of dasatinib monotherapy in patients with relapsed CLL showed a 20% ORR and reported myelosuppression as the major toxicity [86]. However, another phase 2 study of single-agent dasatinib showed a lack of efficacy in heavily pretreated CLL patients, with an only ORR of 6% and a high incidence of neutropenia [87]. Bafetinib, another Lyn kinase inhibitor, also showed efficacy in patients with relapsed/refractory B-CLL in a phase 2 trial [88].

### Hematopoietic stem cell transplantation (HSCT)

Both autologous HSCT (auto-HSCT) and allogeneic HSCT (allo-HSCT) have been increasingly used to treat relapsed or refractory CLL. Auto-HSCT, which solely relies on dose intensity, does not yield better results than modern chemoimmunotherapy. Results of a phase 3 randomized trial of autografting in CLL versus observation for 223 responding patients after first- or second-line treatment indicated that consolidating autografting reduced the risk of progression by more than 50% but had no effect on OS in CLL [89]. Although early treatment intensification including auto-HSCT could provide effective disease control in poor-risk CLL, its clinical benefit compared to FCR regimens remained uncertain [90].

Allo-HSCT has been proven to be the only potentially curative treatment for relapsed CLL patients with fludarabine-refractory disease or a 17p deletion, leading to long-term survival [91,92]. However, myeloablative allo-HSCT showed unacceptable toxicity and mortality in CLL patients [93,94]. Reduced-intensity conditioning (RIC) regimens decrease high transplant-related mortality resulted from severe graft-versus-host disease and infections. The improvement of RIC regimens allows allo-HSCT administrated in older patients and younger patients with co-morbidity [95]. In general, depending on the conditioning regimen and follow-up, RIC allo-HCT was associated with a 11% to 34% nonrelapse mortality, a 34% to 67% PFS, and a 48% to 72% OS [96]. Published literature supports the use of RIC allo-HCT for patients who fulfill acceptable consensus criteria for hematopoietic stem cell allografting, once a suitable donor is identified [97]. In a feasibility analysis of patients with CLL and 17p deletion, Yvonne Hsu et al. reviewed nonmyeloabltive allo-HSCT outcomes for 17p deletion CLL patients transplanted between 2005 and 2010. With a median follow-up of 18 months (range, 3–60), the 2-year OS and PFS rates were 62% and 38%, respectively. Chemosensitivity was associated with significantly higher PFS (73% versus 12%, *P =* 0.02) and a trend for higher OS (91% versus 45%, *P =* 0.09). Nonmyeloabltive allo-HSCT is more effective in 17p deletion CLL patients when recipients have chemosensitive disease [98].

## Conclusion and future directions

Numerous treatment strategies for CLL have emerged over the years, from the use of chlorambucil to RIC allo-HSCT, which revolutionized the treatment and prognosis of CLL. Treatment must be flexible and tailored for different patient groups [3]. The combination of chemotherapy with monoclonal antibodies has further improved response rates and is now the preferred regimen for younger patients requiring treatment. Nonetheless, allo-HSCT remains the only curative option. RIC allo-HSCT must be strongly considered whenever feasible. Newer antibodies such as anti-CD20 antibodies veltuzumab, AME-133v, LFB-R603 and small modular immunopharmaceutical (SMIP) TRU-015, anti-CD37 SMIP TRU-016, anti-CD40 antibodies dacetuzumab (SGN-40), lucatumumab (HCD122), and novel targeted therapeutic agents such as heat shock protein 90 inhibitors SNX-7081, 17-AAG and 17-DMAG, histone deacetylase inhibitors depsipeptide, MS-275, valproic acid, belinostat, MGCD0103, p53 inhibitor cenersen, murine double minute 2 inhibitor RO5045337, and mammalian target of rapamycin (mTOR) inhibitor RAD001, are being investigated in early-phase clinical trials in patients with CLL or NHL [99-111]. Such agents with novel mechanisms of action and targeting different pathways will open a new way to the future treatment for CLL.

## Abbreviations

ATM, Ataxia telangiectasia mutated; BCR, B-cell receptor; BF-ref, Fludarabine-refractory chronic lymphocytic leukemia with bulky (>5 cm) lymphadenopathy; CC, Cladribine and cyclophosphamide; CCR, Clinical complete responses; CDK, Cyclin-dependent kinase; CFAR, Cyclophosphamide, fludarabine, alemtuzumab, and rituximab; CHOP-R, Cyclophosphamide, adriamycin, vincristine and prednisone plus rituximab; CLL, Chronic lymphocytic leukemia; CMC, Cladribine, cyclphosphamide plus mitoxantroneshowed; CR, Complete responses; CRi, Complete responses with incomplete blood recovery; FA-ref, Refractory to fludarabine and alemtuzumab; FBR, Fludarabine, bendamustine, and rituximab; FC, Fludarabine and cyclophosphamide; FCR, Fludarabine, cyclophosphamide, and rituximab; FDA, Food and drug administration; HDMP, High-dose methylprednisolone; HSCT, Hematopoietic stem cell transplantation; IGHV, Immunoglobulin heavy chain variable region genes; MRD, Minimal residual disease; mTOR, Mammalian target of rapamycin; nPR, Nodular partial response; ORR, Overall response rate; OS, Overall survival; PCR, Pentostatin, cyclophosphamide, and rituximab; PD, Progressive disease; PFS, Progression-free survival; PI3K, Phosphatidylinositol-3-kinase; PR, Partial response; RIC, Reduced-intensity conditioning; SD, Stable disease; SLL, Small lymphocytic lymphoma; SMIP, Small modular immunopharmaceutical; ZAP70, Zeta-chain-associated protein kinase 70.

## Competing interests

The authors declare that they have no competing interests.

## Authors’ contributions

Both authors participated in drafting and editing the manuscript. Both authors read and approved the final manuscript.

## References

[B1] Dighiero G Hamblin TJ Chronic lymphocytic leukaemia Lancet 2008 371 1017 1029 10.1016/S0140-6736(08)60456-0 18358929

[B2] Zenz T Mertens D Kuppers R Dohner H Stilgenbauer S From pathogenesis to treatment of chronic lymphocytic leukaemia Nat Rev Cancer 2010 10 37 50 19956173 10.1038/nrc2764

[B3] Maddocks KJ Lin TS Update in the management of chronic lymphocytic leukemia J Hematol Oncol 2009 2 29 10.1186/1756-8722-2-29 19619273 PMC2723130

[B4] Rai KR Peterson BL Appelbaum FR Kolitz J Elias L Shepherd L Hines J Threatte GA Larson RA Cheson BD Schiffer CA Fludarabine compared with chlorambucil as primary therapy for chronic lymphocytic leukemia N Engl J Med 2000 343 1750 1757 10.1056/NEJM200012143432402 11114313

[B5] Eichhorst BF Busch R Stilgenbauer S Stauch M Bergmann MA Ritgen M Kranzhofer N Rohrberg R Soling U Burkhard O First-line therapy with fludarabine compared with chlorambucil does not result in a major benefit for elderly patients with advanced chronic lymphocytic leukemia Blood 2009 114 3382 3391 10.1182/blood-2009-02-206185 19605849

[B6] Leoni LM Bailey B Reifert J Bendall HH Zeller RW Corbeil J Elliott G Niemeyer CC Bendamustine (Treanda) displays a distinct pattern of cytotoxicity and unique mechanistic features compared with other alkylating agents Clin Cancer Res 2008 14 309 317 10.1158/1078-0432.CCR-07-1061 18172283

[B7] Knauf WU Lissichkov T Aldaoud A Liberati A Loscertales J Herbrecht R Juliusson G Postner G Gercheva L Goranov S Phase III randomized study of bendamustine compared with chlorambucil in previously untreated patients with chronic lymphocytic leukemia J Clin Oncol 2009 27 4378 4384 10.1200/JCO.2008.20.8389 19652068

[B8] Kolibaba KS Joshi AD Sterchele JA Forsyth M Alwon E Beygi H Kennealey GT Demographics, Treatment Patterns, Safety, and Real-World Effectiveness in Patients > =70 Years of Age with Chronic Lymphocytic Leukemia Receiving Bendamustine with or without Rituximab ASH Annual Meeting Abstracts 2011 118 3914

[B9] Eichhorst BF Busch R Hopfinger G Pasold R Hensel M Steinbrecher C Siehl S Jager U Bergmann M Stilgenbauer S Fludarabine plus cyclophosphamide versus fludarabine alone in first-line therapy of younger patients with chronic lymphocytic leukemia Blood 2006 107 885 891 16219797 10.1182/blood-2005-06-2395

[B10] Catovsky D Richards S Matutes E Oscier D Dyer MJ Bezares RF Pettitt AR Hamblin T Milligan DW Child JA Assessment of fludarabine plus cyclophosphamide for patients with chronic lymphocytic leukaemia (the LRF CLL4 Trial): a randomised controlled trial Lancet 2007 370 230 239 10.1016/S0140-6736(07)61125-8 17658394

[B11] Flinn IW Neuberg DS Grever MR Dewald GW Bennett JM Paietta EM Hussein MA Appelbaum FR Larson RA Moore DF Jr Tallman MS Phase III trial of fludarabine plus cyclophosphamide compared with fludarabine for patients with previously untreated chronic lymphocytic leukemia: US Intergroup Trial E2997 J Clin Oncol 2007 25 793 798 10.1200/JCO.2006.08.0762 17283364

[B12] Stilgenbauer S Eichhorst BF Busch R Zenz T Winkler D Buhler A Goede V Wendtner CM Lichter P Emmerich B Biologic and Clinical Markers for Outcome after Fludarabine (F) or F Plus Cyclophosphamide (FC) - Comprehensive Analysis of the CLL4 Trial of the GCLLSG ASH Annual Meeting Abstracts 2089 2008 112

[B13] Robak T Jamroziak K Gora-Tybor J Stella-Holowiecka B Konopka L Ceglarek B Warzocha K Seferynska I Piszcz J Calbecka M Comparison of cladribine plus cyclophosphamide with fludarabine plus cyclophosphamide as first-line therapy for chronic lymphocytic leukemia: a phase III randomized study by the Polish Adult Leukemia Group (PALG-CLL3 Study) J Clin Oncol 2010 28 1863 1869 10.1200/JCO.2009.25.9630 20212251

[B14] Robak T Blonski JZ Gora-Tybor J Jamroziak K Dwilewicz-Trojaczek J Tomaszewska A Konopka L Ceglarek B Dmoszynska A Kowal M Cladribine alone and in combination with cyclophosphamide or cyclophosphamide plus mitoxantrone in the treatment of progressive chronic lymphocytic leukemia: report of a prospective, multicenter, randomized trial of the Polish Adult Leukemia Group (PALG CLL2) Blood 2006 108 473 479 10.1182/blood-2005-12-4828 16551966

[B15] Robak T Blonski J Jamroziak K Calbecka M Dwilewicz-Trojaczek J Boguradzki P Dmoszynska A Kowal M Kloczko J Piszcz J Cladribine Alone and in Combination with Cyclophosphamide or Cyclophosphamide and Mitoxantrone in Previously Untreated Patients with Chronic Lymphocytic Leukemia (CLL): Long-Term Follow-up of the the Polish Adult Leukemia Group (PALG CLL2) Study ASH Annual Meeting Abstracts 2011 118 3915

[B16] Byrd JC Peterson BL Morrison VA Park K Jacobson R Hoke E Vardiman JW Rai K Schiffer CA Larson RA Randomized phase 2 study of fludarabine with concurrent versus sequential treatment with rituximab in symptomatic, untreated patients with B-cell chronic lymphocytic leukemia: results from Cancer and Leukemia Group B 9712 (CALGB 9712) Blood 2003 101 6 14 10.1182/blood-2002-04-1258 12393429

[B17] Woyach JA Ruppert AS Heerema NA Peterson BL Gribben JG Morrison VA Rai KR Larson RA Byrd JC Chemoimmunotherapy with fludarabine and rituximab produces extended overall survival and progression-free survival in chronic lymphocytic leukemia: long-term follow-up of CALGB study 9712 J Clin Oncol 2011 29 1349 1355 10.1200/JCO.2010.31.1811 21321292 PMC3084002

[B18] Kay NE Geyer SM Call TG Shanafelt TD Zent CS Jelinek DF Tschumper R Bone ND Dewald GW Lin TS Combination chemoimmunotherapy with pentostatin, cyclophosphamide, and rituximab shows significant clinical activity with low accompanying toxicity in previously untreated B chronic lymphocytic leukemia Blood 2007 109 405 411 10.1182/blood-2006-07-033274 17008537 PMC1785105

[B19] Kay NE Wu W Kabat B LaPlant B Lin TS Byrd JC Jelinek DF Grever MR Zent CS Call TG Shanafelt TD Pentostatin and rituximab therapy for previously untreated patients with B-cell chronic lymphocytic leukemia Cancer 2010 116 2180 2187 20187101 10.1002/cncr.25028 PMC2919331

[B20] Reynolds C Di Bella N Lyons RM Hyman W Richards DA Robbins GJ Vellek M Boehm KA Zhan F Asmar L A Phase III trial of fludarabine, cyclophosphamide, and rituximab vs. pentostatin, cyclophosphamide, and rituximab in B-cell chronic lymphocytic leukemia Invest New Drugs 2012 30 1232 1240 10.1007/s10637-011-9737-y 21922186

[B21] Wierda WG Balakrishnan K Ferrajoli A Kadia T Cortes JE O'Brien S Burger JA Tambaro FP Jalayer A Lerner S A Phase I/II Trial of Fludarabine, Bendamustine, and Rituximab (FBR) Chemoimmunotherapy for Previously Treated Patients with CLL ASH Annual Meeting Abstracts 2011 118 3901

[B22] Weide R Feiten S Friesenhahn V Heymanns J Kleboth K Thomalla J van Roye C Koppler H Retherapy with Bendamustine-Containing Regimens in Patients with Relapsed/Refractory CLL and Indolent Lymphomas Achieves High Response Rates ASH Annual Meeting Abstracts 2011 118 1615

[B23] Bosch F Abrisqueta P Villamor N Terol MJ Gonzalez-Barca E Gonzalez M Ferra C Abella E Delgado J Garcia-Marco JA Rituximab Maintenance In Patients with Chronic Lymphocytic Leukemia (CLL) After Upfront Treatment with Rituximab Plus Fludarabine, Cyclophosphamide, and Mitoxantrone (R-FCM): Final Results of a Multicenter Phase II Trial On Behalf of the Spanish CLL Study Group (GELLC) ASH Annual Meeting Abstracts 2011 118 293

[B24] Jenke P Eichhorst B Busch R Anheier N Duehrsen U Duerig J Dreyling MH Bergmann M Goebeler ME Hurtz HJ Cyclophosphamide, Adriamycin, Vincristine and Prednisone Plus Rituximab (CHOP-R) in Fludarabine (F) Refractory Chronic Lymphocytic Leukemia (CLL) or CLL with Autoimmune Cytopenia (AIC) or Richter's Transformation (RT): Final Analysis of a Phase II Study of the German CLL Study Group ASH Annual Meeting Abstracts 2011 118 2860

[B25] Hillmen P Skotnicki AB Robak T Jaksic B Dmoszynska A Wu J Sirard C Mayer J Alemtuzumab compared with chlorambucil as first-line therapy for chronic lymphocytic leukemia J Clin Oncol 2007 25 5616 5623 10.1200/JCO.2007.12.9098 17984186

[B26] Cortelezzi A Gritti G Laurenti L Cuneo A Ciolli S Di Renzo N Musto P Mauro FR Cascavilla N Falchi L An Italian retrospective study on the routine clinical use of low-dose alemtuzumab in relapsed/refractory chronic lymphocytic leukaemia patients Br J Haematol 2012 156 481 489 10.1111/j.1365-2141.2011.08965.x 22150204

[B27] Gritti G Reda G Maura F Piciocchi A Baldini L Molica S Neri A Cortelezzi A Low dose alemtuzumab in patients with fludarabine-refractory chronic lymphocytic leukemia Leuk Lymphoma 2012 53 424 429 10.3109/10428194.2011.623258 21919823

[B28] Stilgenbauer S Cymbalista F Leblond V Delmer A Winkler D Buhler A Zenz T Mack S Busch R Hinke A Alemtuzumab Plus Oral Dexamethasone. Followed by Alemtuzumab Maintenance or Allogeneic Transplantation in Ultra High-Risk CLL: Interim Analysis of a Phase II Study of the GCLLSG and fcgcll/MW. ASH Annual Meeting Abstracts 2011 118 2854

[B29] Geisler CH van 't Veer MM van Putten W Jurlander J Walewski J Tjonnfjord G Itala-Remes M Kimby E Kozak T Polliack A Immunochemotherapy with Low-Dose Subcutaneous Alemtuzumab (A) Plus Oral Fludarabine and Cyclophosphamide (FC) Is Safe and Induces More and Deeper Complete Remissions in Untreated Patients with High-Risk Chronic Lymphocytic Leukemia (CLL) Than Chemotherapy with FC Alone. An Early Analysis of the Randomized Phase-III HOVON68 CLL Trial ASH Annual Meeting Abstracts 2011 118 290

[B30] Badoux XC Keating MJ Wang X O'Brien SM Ferrajoli A Faderl S Burger J Koller C Lerner S Kantarjian H Wierda WG Cyclophosphamide, fludarabine, alemtuzumab, and rituximab as salvage therapy for heavily pretreated patients with chronic lymphocytic leukemia Blood 2011 118 2085 2093 10.1182/blood-2011-03-341032 21670470 PMC4123326

[B31] Zent CS LaPlant BR Link BK Call TG Shanafelt TD Bowen DA Kay N Weiner GJ Witzig TE Pentostatin, Alemtuzumab, and Low Dose Rituximab Is Effective Therapy for Relapsed/Refractory Chronic Lymphocytic Leukemia/Small Lymphocytic Lymphoma (CLL) ASH Annual Meeting Abstracts 2011 118 1790

[B32] Delmer A Tournilhac O Lepretre S Cazin B Feugier P Dreyfus B Mahe B Leporrier M Michallet A-S Jaccard A Consolidation Therapy with Subcutaneous Alemtuzumab After Induction Treatment with Oral FC (Fludarabine and Cyclophosphamide) in Previously Untreated Patients Aged 65–70 Years with Advanced Stage Chronic Lymphocytic Leukemia (CLL): Final Results of a Phase II Study From the FCGCLL/WM ASH Annual Meeting Abstracts 2011 118 3900

[B33] Castro JE Ariza-Serrano LM Barajas-Gamboa JS Diaz-Perez JA James DF Ale-Ali A Kipps TJ Eradication of Minimal Residual Disease Using Alemtuzumab Consolidation After High-Dose Methyl-Prednisolone Plus Rituximab (HDMP-R) Is Safe, Effective and Induces Long Term Remission in Chronic Lymphocytic Leukemia ASH Annual Meeting Abstracts 2011 118 2866

[B34] Jones JA Stark A Zhao W Lin TS Rai KR Marcucci G Peterson B Larson RA Heerema NA Byrd JC Alemtuzumab Consolidation Does Not Improve Outcome for CLL Patients with High Risk Genomic Features on Successive CALGB Trials ASH Annual Meeting Abstracts 2011 118 1791

[B35] Wierda WG Kipps TJ Mayer J Stilgenbauer S Williams CD Hellmann A Robak T Furman RR Hillmen P Trneny M Ofatumumab as single-agent CD20 immunotherapy in fludarabine-refractory chronic lymphocytic leukemia J Clin Oncol 2010 28 1749 1755 10.1200/JCO.2009.25.3187 20194866 PMC4979101

[B36] Wierda WG Kipps TJ Mayer J Robak T Dyer MJ Furman RR Hillmen P Stilgenbauer S Williams CD Trneny M Final Analysis From the International Trial of Single-Agent Ofatumumab In Patients with Fludarabine-Refractory Chronic Lymphocytic Leukemia ASH Annual Meeting Abstracts 2010 116 921

[B37] Flinn IW Harwin WN Macias-Perez IM Tucker PS Waterhouse DM Papish SW Jones JA Hainsworth JD Byrd JC A Phase II Trial of Ofatumumab for Older Patients and Patients Who Refuse Fludarabine-Based Regimens with Previously Untreated Chronic Lymphocytic Leukemia or Small Lymphocytic Lymphoma ASH Annual Meeting Abstracts 2011 118 3912

[B38] Wierda WG Kipps TJ Durig J Griskevicius L Stilgenbauer S Mayer J Smolej L Hess G Griniute R Hernandez-Ilizaliturri FJ Chemoimmunotherapy with O-FC in previously untreated patients with chronic lymphocytic leukemia Blood 2011 117 6450 6458 10.1182/blood-2010-12-323980 21498674 PMC4916561

[B39] Shanafelt TD Lanasa MC Zent CS Leis JF Call TG LaPlant BR Tun H Bowen DA Jelinek DF Hanson CA Kay N Ofatumumab Based Chemoimmunotherapy (CIT) for Patients with Previously Untreated CLL ASH Annual Meeting Abstracts 2011 118 3898

[B40] Byrd JC O'Brien S Flinn IW Kipps TJ Weiss M Rai K Lin TS Woodworth J Wynne D Reid J Phase 1 study of lumiliximab with detailed pharmacokinetic and pharmacodynamic measurements in patients with relapsed or refractory chronic lymphocytic leukemia Clin Cancer Res 2007 13 4448 4455 10.1158/1078-0432.CCR-06-1463 17671129

[B41] Byrd JC Kipps TJ Flinn IW Castro J Lin TS Wierda W Heerema N Woodworth J Hughes S Tangri S Phase 1/2 study of lumiliximab combined with fludarabine, cyclophosphamide, and rituximab in patients with relapsed or refractory chronic lymphocytic leukemia Blood 2010 115 489 495 10.1182/blood-2009-08-237727 19843887 PMC2810983

[B42] Jaglowski SM Alinari L Lapalombella R Muthusamy N Byrd JC The clinical application of monoclonal antibodies in chronic lymphocytic leukemia Blood 2010 116 3705 3714 10.1182/blood-2010-04-001230 20610811 PMC2981531

[B43] Umana P Moessner E Bruenker P Unsin G Puentener U Suter T Grau R Schmidt C Gerdes C Nopora A Novel 3rd Generation Humanized Type II CD20 Antibody with Glycoengineered Fc and Modified Elbow Hinge for Enhanced ADCC and Superior Apoptosis Induction ASH Annual Meeting Abstracts 2006 108 229

[B44] Bologna L Gotti E Manganini M Rambaldi A Intermesoli T Introna M Golay J Mechanism of action of type II, glycoengineered, anti-CD20 monoclonal antibody GA101 in B-chronic lymphocytic leukemia whole blood assays in comparison with rituximab and alemtuzumab J Immunol 2011 186 3762 3769 10.4049/jimmunol.1000303 21296976

[B45] Morschhauser F Cartron G Lamy T Milpied N-J Thieblemont C Tilly H Weisser M Birkett J Salles GA Phase I Study of RO5072759 (GA101) in Relapsed/Refractory Chronic Lymphocytic Leukemia ASH Annual Meeting Abstracts 2009 114 884

[B46] Lacy MQ Allred JB Gertz MA Hayman SR Short KD Buadi F Dispenzieri A Kumar S Greipp PR Lust JA Pomalidomide plus low-dose dexamethasone in myeloma refractory to both bortezomib and lenalidomide: comparison of 2 dosing strategies in dual-refractory disease Blood 2011 118 2970 2975 10.1182/blood-2011-04-348896 21690557 PMC3291492

[B47] Begna KH Pardanani A Mesa R Litzow MR Hogan WJ Hanson CA Tefferi A Long-term outcome of pomalidomide therapy in myelofibrosis Am J Hematol 2012 87 66 68 10.1002/ajh.22233 22081489

[B48] Kotla V Goel S Nischal S Heuck C Vivek K Das B Verma A Mechanism of action of lenalidomide in hematological malignancies J Hematol Oncol 2009 2 36 10.1186/1756-8722-2-36 19674465 PMC2736171

[B49] Badoux XC Keating MJ Wen S Lee BN Sivina M Reuben J Wierda WG O'Brien SM Faderl S Kornblau SM Lenalidomide as initial therapy of elderly patients with chronic lymphocytic leukemia Blood 2011 118 3489 3498 10.1182/blood-2011-03-339077 21725050 PMC4123422

[B50] James DF Brown JR Werner L Wierda WG Barrientos JC Castro J Greaves A Rassenti LZ Rai KR Neuberg D Kipps TJ Lenalidomide and Rituximab for the Initial Treatment of Patients with Chronic Lymphocytic Leukemia (CLL) A Multicenter Study of the CLL Research Consortium ASH Annual Meeting Abstracts 2011 118 291

[B51] Badoux XC Keating MJ O'Brien S Wierda WG Faderl S Estrov Z Pasia M Lerner S Sargent RL Kantarjian HM Ferrajoli A Final Analysis of a Phase 2 Study of Lenalidomide and Rituximab in Patients with Relapsed or Refractory Chronic Lymphocytic Leukemia (CLL) ASH Annual Meeting Abstracts 2011 118 980

[B52] Ferrajoli A O'Brien S Wierda WG Faderl S Badoux X Estrov Z Schlette E Smith SC Ayala AB Falchi L Combination Therapy with Ofatumumab and Lenalidomide in Patients with Relapsed Chronic Lymphocytic Leukemia (CLL): Results of a Phase II Trial ASH Annual Meeting Abstracts 2011 118 1788

[B53] Shanafelt TD Tun H Hanson CA Zent CS Leis JF Call TG LaPlant BR Bowen D Pavey ES Jelinek DF Kay N Lenalidomide Consolidation Appears to Prolong Time to Retreatment After First-Line Chemoimmunotherapy for Patients with Previously Untreated CLL ASH Annual Meeting Abstracts 2011 118 3899

[B54] Pozadzides JV Keating MJ Wierda WG O'Brien S Burger JA Jorgensen JL Calin S Wang S Lerner S Ferrajoli A Initial Experience with Lenalidomide As Consolidation Treatment in Patients with Chronic Lymphocytic Leukemia and Residual Disease After Chemotherapy ASH Annual Meeting Abstracts 2011 118 3907

[B55] Bosanquet AG McCann SR Crotty GM Mills MJ Catovsky D Methylprednisolone in advanced chronic lymphocytic leukaemia: rationale for, and effectiveness of treatment suggested by DiSC assay Acta Haematol 1995 93 73 79 10.1159/000204115 7543720

[B56] Thornton PD Hamblin M Treleaven JG Matutes E Lakhani AK Catovsky D High dose methyl prednisolone in refractory chronic lymphocytic leukaemia Leuk Lymphoma 1999 34 167 170 10350345 10.3109/10428199909083393

[B57] Thornton PD Matutes E Bosanquet AG Lakhani AK Grech H Ropner JE Joshi R Mackie PH Douglas ID Bowcock SJ Catovsky D High dose methylprednisolone can induce remissions in CLL patients with p53 abnormalities Ann Hematol 2003 82 759 765 10.1007/s00277-003-0710-5 14551737

[B58] Castro JE Sandoval-Sus JD Bole J Rassenti L Kipps TJ Rituximab in combination with high-dose methylprednisolone for the treatment of fludarabine refractory high-risk chronic lymphocytic leukemia Leukemia 2008 22 2048 2053 10.1038/leu.2008.214 18754025 PMC5289283

[B59] Castro JE James DF Sandoval-Sus JD Jain S Bole J Rassenti L Kipps TJ Rituximab in combination with high-dose methylprednisolone for the treatment of chronic lymphocytic leukemia Leukemia 2009 23 1779 1789 10.1038/leu.2009.133 19693094 PMC2761991

[B60] Bowen DA Call TG Jenkins GD Zent CS Schwager SM Van Dyke DL Jelinek DF Kay NE Shanafelt TD Methylprednisolone-rituximab is an effective salvage therapy for patients with relapsed chronic lymphocytic leukemia including those with unfavorable cytogenetic features Leuk Lymphoma 2007 48 2412 2417 10.1080/10428190701724801 18067017

[B61] Pileckyte R Jurgutis M Valceckiene V Stoskus M Gineikiene E Sejoniene J Degulys A Zvirblis T Griskevicius L Dose-dense high-dose methylprednisolone and rituximab in the treatment of relapsed or refractory high-risk chronic lymphocytic leukemia Leuk Lymphoma 2011 52 1055 1065 10.3109/10428194.2011.562572 21599591

[B62] Castro JE Barajas-Gamboa JS Melo-Cardenas J Paz RN Bernal-Corzo C Ale-Ali A James DF Kipps TJ Ofatumumab and High-Dose Methylprednisolone Is An Effective Salvage Treatment for Heavily Pretreated, Unfit or Refractory Patients with Chronic Lymphocytic Leukemia: Single Institution Experience. ASH Annual Meeting Abstracts 2010 116 4638

[B63] Lin TS Ruppert AS Johnson AJ Fischer B Heerema NA Andritsos LA Blum KA Flynn JM Jones JA Hu W Phase II study of flavopiridol in relapsed chronic lymphocytic leukemia demonstrating high response rates in genetically high-risk disease J Clin Oncol 2009 27 6012 6018 10.1200/JCO.2009.22.6944 19826119 PMC2793044

[B64] Lanasa MC Andritsos L Brown JR Gabrilove J Caligaris-Cappio F Larson R Kipps TJ Leblond V Milligan D Janssens A Interim Analysis of EFC6663, a Multicenter Phase 2 Study of Alvocidib (flavopiridol), Demonstrates Clinical Responses Among Patients with Fludarabine Refractory CLL. ASH Annual Meeting Abstracts 2010 116 58

[B65] Woyach JA Ruppert AS Blum KA Jones JA Flynn JM Johnson AJ Grever MR Byrd JC Heerema NA Response, Progression-Free Survival, and Overall Survival of Patients with Relapsed or Refractory Chronic Lymphocytic Leukemia (CLL) Treated with Flavopiridol: Impact of Poor Risk Cytogenetic Abnormalities ASH Annual Meeting Abstracts 2010 116 2456

[B66] Stephens DM Ruppert AS Blum K Jones J Flynn JM Johnson AJ Ji J Phelps MA Grever MR Byrd JC Flavopiridol treatment of patients aged 70 or older with refractory or relapsed chronic lymphocytic leukemia is a feasible and active therapeutic approach Haematologica 2012 97 423 427 10.3324/haematol.2011.047324 22271900 PMC3291598

[B67] Blum KA Wei L Jones JA Andritsos LA Flynn JM Heerema NA Yang X Rozewski D Phelps M Johnson AJ Activity of Combined Flavopiridol and Lenalidomide in Patients with Cytogenetically High Risk Chronic Lymphocytic Leukemia (CLL): Updated Results of a Phase I Trial ASH Annual Meeting Abstracts 2011 118 3910

[B68] Flynn JM Jones JA Andritsos L Blum KA Johnson AJ Hessler J Wiley E Poon J Small K Statkevich P Update on the Phase I Study of the Cyclin Dependent Kinase Inhibitor Dinaciclib (SCH 727965) In Patients with Relapsed or Refractory Chronic Lymphocytic Leukemia (CLL): Confirmation of Clinical Activity and Feasibility of Long-Term Administration ASH Annual Meeting Abstracts 2010 116 1396

[B69] Roberts AW Seymour JF Brown JR Wierda WG Kipps TJ Carney D Xiong H Cui Y Busman T Enschede S An Ongoing Phase 1/2a Study of ABT-263; Pharmacokinetics (PK), Safety and Anti-Tumor Activity in Patients (pts) with Relapsed or Refractory Chronic Lymphocytic Leukemia (CLL) ASH Annual Meeting Abstracts 2009 114 883

[B70] Roberts AW Seymour JF Brown JR Wierda WG Kipps TJ Khaw SL Carney DA He SZ Huang DC Xiong H Substantial susceptibility of chronic lymphocytic leukemia to BCL2 inhibition: results of a phase I study of navitoclax in patients with relapsed or refractory disease J Clin Oncol 2012 30 488 496 10.1200/JCO.2011.34.7898 22184378 PMC4979082

[B71] Kipps TJ Swinnen LJ Wierda WG Jones JA Coutre SE Smith MR Yang J Cui Y Busman T Enschede S Humerickhouse R Navitoclax (ABT-263) Plus Fludarabine/Cyclophosphamide/Rituximab (FCR) or Bendamustine/Rituximab (BR): A Phase 1 Study in Patients with Relapsed/Refractory Chronic Lymphocytic Leukemia (CLL) ASH Annual Meeting Abstracts 2011 118 3904

[B72] O'Brien S Moore JO Boyd TE Larratt LM Skotnicki AB Koziner B Chanan-Khan AA Seymour JF Gribben J Itri LM Rai KR 5-year survival in patients with relapsed or refractory chronic lymphocytic leukemia in a randomized, phase III trial of fludarabine plus cyclophosphamide with or without oblimersen J Clin Oncol 2009 27 5208 5212 10.1200/JCO.2009.22.5748 19738118 PMC5321078

[B73] Balakrishnan K Burger JA Wierda WG Gandhi V AT-101 induces apoptosis in CLL B cells and overcomes stromal cell-mediated Mcl-1 induction and drug resistance Blood 2009 113 149 153 10.1182/blood-2008-02-138560 18836097 PMC2614629

[B74] O'Brien SM Claxton DF Crump M Faderl S Kipps T Keating MJ Viallet J Cheson BD Phase I study of obatoclax mesylate (GX15-070), a small molecule pan-Bcl-2 family antagonist, in patients with advanced chronic lymphocytic leukemia Blood 2009 113 299 305 18931344 10.1182/blood-2008-02-137943 PMC4968372

[B75] Durig J Duhrsen U Klein-Hitpass L Worm J Hansen JB Orum H Wissenbach M The novel antisense Bcl-2 inhibitor SPC2996 causes rapid leukemic cell clearance and immune activation in chronic lymphocytic leukemia Leukemia 2011 25 638 647 10.1038/leu.2010.322 21358717

[B76] Hoellenriegel J Meadows SA Sivina M Wierda WG Kantarjian H Keating MJ Giese N O'Brien S Yu A Miller LL The phosphoinositide 3'-kinase delta inhibitor, CAL-101, inhibits B-cell receptor signaling and chemokine networks in chronic lymphocytic leukemia Blood 2011 118 3603 3612 10.1182/blood-2011-05-352492 21803855 PMC4916562

[B77] Furman RR Byrd JC Brown JR Coutre SE Benson DM Wagner-Johnston ND Flinn IW Kahl BS Spurgeon SE Lannutti B CAL-101, An Isoform-Selective Inhibitor of Phosphatidylinositol 3-Kinase P110{delta}, Demonstrates Clinical Activity and Pharmacodynamic Effects In Patients with Relapsed or Refractory Chronic Lymphocytic Leukemia ASH Annual Meeting Abstracts 2010 116 55

[B78] Sharman J de Vos S Leonard JP Furman RR Coutre SE Flinn IW Schreeder MT Barrientos JC Wagner-Johnston ND Boyd T A Phase 1 Study of the Selective Phosphatidylinositol 3-Kinase-Delta (PI3K{delta}) Inhibitor, CAL-101 (GS-1101), in Combination with Rituximab and/or Bendamustine in Patients with Relapsed or Refractory Chronic Lymphocytic Leukemia (CLL) ASH Annual Meeting Abstracts 2011 118 1787

[B79] Brown JR Davids MS Rodon J Abrisqueta P DeCillis AP Rockich K Egile C Kelly A Xu Y Lager J Awan FT Phase I Trial of SAR245408 (S08), a Pan-Phosphatidylinositol 3 Kinase (PI3K) Inhibitor, in Patients with Chronic Lymphocytic Leukemia (CLL) and Lymphoma ASH Annual Meeting Abstracts 2011 118 2683

[B80] de Rooij MF Kuil A Geest CR Eldering E Chang BY Buggy JJ Pals ST Spaargaren M The clinically active BTK inhibitor PCI-32765 targets B-cell receptor- and chemokine-controlled adhesion and migration in chronic lymphocytic leukemia Blood 2012 119 2590 2594 10.1182/blood-2011-11-390989 22279054

[B81] Byrd JC Blum KA Burger JA Coutre SE Sharman JP Furman RR Flinn IW Grant BW Richards DA Zhao W Activity and tolerability of the Bruton's tyrosine kinase (Btk) inhibitor PCI-32765 in patients with chronic lymphocytic leukemia/small lymphocytic lymphoma (CLL/SLL): Interim results of a phase Ib/II study ASCO Meeting Abstracts 2011 29 6508

[B82] O'Brien S Burger JA Blum KA Furman RR Coutre SE Sharman J Flinn IW Grant B Heerema NA Johnson AJ The Bruton's Tyrosine Kinase (BTK) Inhibitor PCI-32765 Induces Durable Responses in Relapsed or Refractory (R/R) Chronic Lymphocytic Leukemia/Small Lymphocytic Lymphoma (CLL/SLL): Follow-up of a Phase Ib/II Study ASH Annual Meeting Abstracts 2011 118 983

[B83] Friedberg JW Sharman J Sweetenham J Johnston PB Vose JM Lacasce A Schaefer-Cutillo J De Vos S Sinha R Leonard JP Inhibition of Syk with fostamatinib disodium has significant clinical activity in non-Hodgkin lymphoma and chronic lymphocytic leukemia Blood 2010 115 2578 2585 10.1182/blood-2009-08-236471 19965662 PMC2852362

[B84] Quiroga MP Balakrishnan K Kurtova AV Sivina M Keating MJ Wierda WG Gandhi V Burger JA B-cell antigen receptor signaling enhances chronic lymphocytic leukemia cell migration and survival: specific targeting with a novel spleen tyrosine kinase inhibitor, R406 Blood 2009 114 1029 1037 10.1182/blood-2009-03-212837 19491390 PMC4916941

[B85] Hoellenriegel J Coffey GP Sinha U Pandey A Sivina M Ferrajoli A Ravandi F Wierda WG O'Brien S Keating MJ Burger JA Selective, novel spleen tyrosine kinase (Syk) inhibitors suppress chronic lymphocytic leukemia B-cell activation and migration Leukemia 2012 26 1576 1583 10.1038/leu.2012.24 22362000 PMC5459370

[B86] Amrein PC Attar EC Takvorian T Hochberg EP Ballen KK Leahy KM Fisher DC Lacasce AS Jacobsen ED Armand P Phase II study of dasatinib in relapsed or refractory chronic lymphocytic leukemia Clin Cancer Res 2011 17 2977 2986 10.1158/1078-0432.CCR-10-2879 21402714 PMC3108904

[B87] Al-Ameri AM Badoux X Ferrajoli A Wierda WG Fayad L Estrov Z Bickel S Keating MJ O'Brien S Phase II Study of Dasatinib In Patients with Relapsed Chronic Lymphocytic Leukemia ASH Annual Meeting Abstracts 2010 116 4488

[B88] Kadia T Delioukina ML Kantarjian HM Keating MJ Wierda WG Burger JA Wieland S Levitt D A Pilot Phase II Study of the Lyn Kinase Inhibitor Bafetinib in Patients with Relapsed or Refractory B Cell Chronic Lymphocytic Leukemia ASH Annual Meeting Abstracts 2011 118 2858

[B89] Michallet M Dreger P Sutton L Brand R Richards S van Os M Sobh M Choquet S Corront B Dearden C Autologous hematopoietic stem cell transplantation in chronic lymphocytic leukemia: results of European intergroup randomized trial comparing autografting versus observation Blood 2011 117 1516 1521 10.1182/blood-2010-09-308775 21106985

[B90] Dreger P Dohner H McClanahan F Busch R Ritgen M Greinix H Fink AM Knauf W Stadler M Pfreundschuh M Early autologous stem cell transplantation for chronic lymphocytic leukemia: long-term follow-up of the German CLL Study Group CLL3 trial Blood 2012 119 4851 4859 10.1182/blood-2011-09-378505 22490331

[B91] Caballero D Garcia-Marco JA Martino R Mateos V Ribera JM Sarra J Leon A Sanz G de la Serna J Cabrera R Allogeneic transplant with reduced intensity conditioning regimens may overcome the poor prognosis of B-cell chronic lymphocytic leukemia with unmutated immunoglobulin variable heavy-chain gene and chromosomal abnormalities (11q- and 17p-) Clin Cancer Res 2005 11 7757 7763 10.1158/1078-0432.CCR-05-0941 16278397

[B92] Moreno C Villamor N Colomer D Esteve J Martino R Nomdedeu J Bosch F Lopez-Guillermo A Campo E Sierra J Montserrat E Allogeneic stem-cell transplantation may overcome the adverse prognosis of unmutated VH gene in patients with chronic lymphocytic leukemia J Clin Oncol 2005 23 3433 3438 10.1200/JCO.2005.04.531 15809449

[B93] Pavletic SZ Khouri IF Haagenson M King RJ Bierman PJ Bishop MR Carston M Giralt S Molina A Copelan EA Unrelated donor marrow transplantation for B-cell chronic lymphocytic leukemia after using myeloablative conditioning: results from the Center for International Blood and Marrow Transplant research J Clin Oncol 2005 23 5788 5794 10.1200/JCO.2005.03.962 16043827

[B94] Pavletic ZS Arrowsmith ER Bierman PJ Goodman SA Vose JM Tarantolo SR Stein RS Bociek G Greer JP Wu CD Outcome of allogeneic stem cell transplantation for B cell chronic lymphocytic leukemia Bone Marrow Transplant 2000 25 717 722 10.1038/sj.bmt.1702237 10745256

[B95] Dreger P Allotransplantation for chronic lymphocytic leukemia Hematology / the Education Program of the American Society of Hematology American Society of Hematology Education Program 2009 1 602 609 20008245 10.1182/asheducation-2009.1.602

[B96] Delgado J Milligan DW Dreger P Allogeneic hematopoietic cell transplantation for chronic lymphocytic leukemia: ready for prime time? Blood 2009 114 2581 2588 10.1182/blood-2009-05-206821 19641189

[B97] Kharfan-Dabaja MA Bazarbachi A Hematopoietic stem cell allografting for chronic lymphocytic leukemia: a focus on reduced-intensity conditioning regimens Cancer control: journal of the Moffitt Cancer Center 2012 19 68 75 22143063 10.1177/107327481201900107

[B98] Hsu Y Saliba RM Okoroji G-J o'Brien S Ferrajoli A Abruzzo LV Champlin R Keating MJ Khouri IF Feasibility Analysis of Nonmyeloabltive Allogeneic Stem Cell Transplantation (NST) in Patients with Chronic Lymphocytic Leukemia (CLL) and 17p- Deletion ASH Annual Meeting Abstracts 2011 118 4123

[B99] Milani C Castillo J Veltuzumab, an anti-CD20 mAb for the treatment of non-Hodgkin's lymphoma, chronic lymphocytic leukemia and immune thrombocytopenic purpura Curr Opin Mol Ther 2009 11 200 207 19330725

[B100] Forero-Torres A de Vos S Pohlman BL Pashkevich M Cronier DM Dang NH Carpenter SP Allan BW Nelson JG Slapak CA Results of a phase 1 study of AME-133v (LY2469298), an Fc-engineered humanized monoclonal anti-CD20 antibody, in FcgammaRIIIa-genotyped patients with previously treated follicular lymphoma Clin Cancer Res 2012 18 1395 1403 10.1158/1078-0432.CCR-11-0850 22223529

[B101] Baritaki S Militello L Malaponte G Spandidos DA Salcedo M Bonavida B The anti-CD20 mAb LFB-R603 interrupts the dysregulated NF-kappaB/Snail/RKIP/PTEN resistance loop in B-NHL cells: role in sensitization to TRAIL apoptosis Int J Oncol 2011 38 1683 1694 21455568 10.3892/ijo.2011.984

[B102] Hayden-Ledbetter MS Cerveny CG Espling E Brady WA Grosmaire LS Tan P Bader R Slater S Nilsson CA Barone DS CD20-directed small modular immunopharmaceutical, TRU-015, depletes normal and malignant B cells Clin Cancer Res 2009 15 2739 2746 10.1158/1078-0432.CCR-08-1694 19351771

[B103] Robak T Robak P Smolewski P TRU-016, a humanized anti-CD37 IgG fusion protein for the potential treatment of B-cell malignancies Curr Opin Investig Drugs 2009 10 1383 1390 19943209

[B104] Furman RR Forero-Torres A Shustov A Drachman JG A phase I study of dacetuzumab (SGN-40, a humanized anti-CD40 monoclonal antibody) in patients with chronic lymphocytic leukemia Leuk Lymphoma 2010 51 228 235 10.3109/10428190903440946 20038235

[B105] Byrd JC Kipps TJ Flinn IW Cooper M Odenike O Bendiske J Rediske J Bilic S Dey J Baeck J O'Brien S Phase I study of the anti-CD40 humanized monoclonal antibody lucatumumab (HCD122) in relapsed chronic lymphocytic leukemia Leuk Lymphoma 2012 in press 10.3109/10428194.2012.681655 PMC3808981 22475052

[B106] Best OG Che Y Singh N Forsyth C Christopherson RI Mulligan SP The Hsp90 inhibitor SNX-7081 synergizes with and restores sensitivity to fludarabine in chronic lymphocytic leukemia cells with lesions in the TP53 pathway: a potential treatment strategy for fludarabine refractory disease Leuk Lymphoma 2012 53 1367 1375 10.3109/10428194.2011.647310 22149137

[B107] Hertlein E Wagner AJ Jones J Lin TS Maddocks KJ Towns WH 3rd Goettl VM Zhang X Jarjoura D Raymond CA 17-DMAG targets the nuclear factor-kappaB family of proteins to induce apoptosis in chronic lymphocytic leukemia: clinical implications of HSP90 inhibition Blood 2010 116 45 53 10.1182/blood-2010-01-263756 20351313 PMC2904580

[B108] Tan J Cang S Ma Y Petrillo RL Liu D Novel histone deacetylase inhibitors in clinical trials as anti-cancer agents J Hematol Oncol 2010 3 5 10.1186/1756-8722-3-5 20132536 PMC2827364

[B109] Lanasa MC Davis PH Datto M Li Z Gockerman JP Moore JO Decastro CM Friedman DR Diehl LF Rehder C Phase II study of cenersen, an antisense inhibitor of p53, in combination with fludarabine, cyclophosphamide and rituximab for high-risk chronic lymphocytic leukemia Leuk Lymphoma 2012 53 218 224 10.3109/10428194.2011.610012 21827374

[B110] Yuan Y Liao YM Hsueh CT Mirshahidi HR Novel targeted therapeutics: inhibitors of MDM2. ALK and PARP Journal of hematology & oncology 2011 4 16 10.1186/1756-8722-4-16 21504625 PMC3103487

[B111] Decker T Sandherr M Goetze K Oelsner M Ringshausen I Peschel C A pilot trial of the mTOR (mammalian target of rapamycin) inhibitor RAD001 in patients with advanced B-CLL Ann Hematol 2009 88 221 227 10.1007/s00277-008-0582-9 18704419

